# The topography of rods, cones and intrinsically photosensitive retinal ganglion cells in the retinas of a nocturnal (*Micaelamys namaquensis*) and a diurnal (*Rhabdomys pumilio*) rodent

**DOI:** 10.1371/journal.pone.0202106

**Published:** 2018-08-09

**Authors:** Ingrid van der Merwe, Ákos Lukáts, Veronika Bláhová, Maria K. Oosthuizen, Nigel C. Bennett, Pavel Němec

**Affiliations:** 1 Mammal Research Institute, Department of Zoology and Entomology, University of Pretoria, Pretoria, South Africa; 2 Department of Anatomy, Histology and Embryology, Semmelweis University, Budapest, Hungary; 3 Department of Zoology, Charles University, Prague, Czech Republic; Morehouse School of Medicine, UNITED STATES

## Abstract

We used immunocytochemistry to determine the presence and topographical density distributions of rods, cones, and intrinsically photosensitive retinal ganglion cells (ipRGCs) in the four-striped field mouse (*Rhabdomys pumilio*) and the Namaqua rock mouse (*Micaelamys namaquensis*). Both species possessed duplex retinas that were rod dominated. In *R*. *pumilio*, the density of both cones and rods were high (cone to rod ratio: 1:1.23) and reflected the species’ fundamentally diurnal, but largely crepuscular lifestyle. Similarly, the ratio of cones to rods in *M*. *namaquensis* (1:12.4) reflected its nocturnal lifestyle. Similar rod density peaks were observed (*R*. *pumilio*: ~84467/mm^2^; *M*. *namaquensis*: ~81088/mm^2^), but a density gradient yielded higher values in the central (~56618/mm^2^) rather than in the peripheral retinal region (~32689/mm^2^) in *R*. *pumilio*. Two separate cone types (S-cones and M/L-cones) were identified implying dichromatic color vision in the study species. In *M*. *namaquensis*, both cone populations showed a centro-peripheral density gradient and a consistent S- to M/L-cone ratio (~1:7.8). In *R*. *pumilio*, S cones showed a centro-peripheral gradient (S- to M/L-cone ratio; central: 1:7.8; peripheral: 1:6.8) which appeared to form a visual streak, and a specialized area of M/L-cones (S- to M/L-cone ratio: 1:15) was observed inferior to the optic nerve. The number of photoreceptors per linear degree of visual angle, estimated from peak photoreceptor densities and eye size, were four cones and 15 rods per degree in *M*. *namaquensis* and 11 cones and 12 rods per degree in *R*. *pumilio*. Thus, in nocturnal *M*. *namaquensis* rods provide much finer image sampling than cones, whereas in diurnal/crepuscular *R*. *pumilio* both photoreceptor types provide fine image sampling. IpRGCs were comparably sparse in *R*. *pumilio* (total = 1012) and *M*. *namaquensis* (total = 862), but were homogeneously distributed in *M*. *namaquensis* and densest in the dorso-nasal quadrant in *R*. *pumilio*. The adaptive significance of the latter needs further investigation.

## Introduction

The vertebrate eye and in particular the retina, acts as a mediator between the light environment and the brain of an animal, converting external light stimuli into biological signals (neural impulses). Phototransduction in the retina paves the way for the extraction of two distinct categories of information; one for image-forming vision and the other for a range of non-image-forming responses, most notably the synchronization of the biological clock with the daily light-dark cycle [1,2]. The typical mammalian retina contains rods (for scotopic vision) and two cone types (for photopic vision) namely, S-cones (short wavelength sensitive cones) and M/L-cones (medium- to long wavelength sensitive cones) [3]. While vision originates at the rods and cones, non-image-forming processes are regulated by a small subset of melanopsin positive ganglion cells, i.e. the intrinsically sensitive retinal ganglion cells (ipRGCs) [4,5]. The recognition of the ipRGCs as distinct photoreceptors was first suggested by studies showing normal photoentrainment of the circadian system in mice that lacked all functional rods and cones (*rd/rd cl* mice) [1,6,7]. As a result, it has been concluded in the past that conventional photoreceptors do not contribute to non-image forming vision. Nevertheless, studies have since shown that ipRGCs not only receive input from rods and cones for non-image-forming processes, but that they are also involved in image-forming vision [8–11].

In vertebrates, there is an immense variation in the presence, density and topographical array of retinal photoreceptors [3,12]. Since each photoreceptor type only detects a particular range of the light spectrum, the visual perception of a species is restricted by its photoreceptor composition. This is elegantly demonstrated by the presence of ultraviolet sensitive S-cones in the retinas of several rodents, enabling them to detect light that is simply invisible to the human eye [13–15]. Furthermore, the ratio of cones to rods is often a reliable indicator of the daily activity rhythm of an animal because it reflects visual capacity in different photoenvironments [3,16]. For this reason, cones make up a larger proportion of the total visual-photoreceptor population (TVPP) in many diurnal species, e.g. the Californian ground squirrel (*Spermophilus beecheyi*, 86% cones), Sudanian unstriped grass rat (*Arvicanthis ansorgei*: 33% cones), striped desert mouse (*Lemniscomys barbarus*: 33% cones) and the Nile grass rat (*A*. *niloticus*: 35–40% cones) [17–19]. Conversely, the TVPPs of nocturnal rodents contain comparatively small cone populations; e.g. the Gambian pouched rat (*Cricetomys gambianus*: 0.3–0.5% cones), Emin’s pouched rat (*C*. *emini*: 0.5–0.8% cones), eastern woodrat (*Neotoma floridana*: 1% cones), and in conventional laboratory mice and rats (<3% cones) [20–23]. It should however, be noted that although rods still outnumber cones in most diurnal species, the proportion of cones is much higher in diurnal species compared to nocturnal species. The visual capabilities of an animal are determined not only by the composition of photoreceptors but also by their topographical density arrangement. Retinal specializations such as areae centrales, foveae and visual streaks allow higher visual resolution in parts of the visual field that are important to the particular species. For example, many animals that inhabit environments where there is a clear view of the horizon commonly have horizontal or visual retinal streaks, whereas animals (e.g. arboreal species) from cluttered environments often have areae centrales [24]. Another example includes the bias of S-cones in the ventral retina of many smaller terrestrial mammals, which is suggested to improve the discrimination of objects against an open sky (e.g. screening for aerial predators) [25,26]. Even though several theories seem plausible in explaining the functional significance of different photoreceptor mosaics, much remains to be uncovered regarding how retinal specializations improve vision in specific animals [25].

The present study aimed to characterize the retinal photoreceptor topographies in two South African rodents with disparate lifestyles that inhabit different photoenvironments. For this, the density and topographical arrangement of rods, cones and ipRGCs were investigated and compared by means of immunocytochemistry in the diurnally active four-striped field mouse (*Rhabdomys pumilio*) and the nocturnally active Namaqua rock mouse (*Micaelamys namaquensis*). As its colloquial name indicates, *M*. *namaquensis* prefers rocky environments above other habitat types and frequently finds refuge among rock crevices, whereas *R*. *pumilio* is highly adaptable to a variety of habitats, but typically prefers areas which are covered with grass and consequently avoids open areas [27]. Retinal photoreceptor compositions reflect the temporal activity patterns of the studied species. In both species, the activity rhythm is entrained by the daily light-dark cycle: *M*. *namaquensis* is nearly exclusively active at night and *R*. *pumilio* is fundamentally diurnal, but with marked activity at dusk and dawn [27,28].

## Materials and methods

### Animal and tissue preparation

The project was approved by the Animal Ethics Committee of the University of Pretoria, Pretoria, South Africa (EC063-11). The eyes from adult laboratory-bred *R*. *pumilio* (Sparrman 1784) and wild-caught *M*. *namaquensis* (Smith 1834) were used in this study. *Rhabdomys pumilio* were obtained from a breeding colony at the University of the Witwatersrand (Johannesburg, South Africa), while *M*. *namaquensis* were collected (trapping permit: 001-CPM403-00014) from Goro Game Reserve in the Soutpansberg region (Limpopo Province, South Africa). Before the animals were euthanized and enucleated they were maintained in a climate controlled room (ambient temperature: 25 ± 1°C, relative humidity: ~60%). The animals were maintained individually in semi-transparent plastic cages (58 x 38 x 36 cm) with a layer of wood shavings (± 3 cm thick) and were given a small plastic shelter with tissue paper for nesting material. The mice had *ad libitum* access to water and food, (apple, carrot and seed mix). The seed mix (parrot seed mix; Marlton’s, Durban, South Africa) and water were topped up and the fresh food was replaced at random times every second or third day. The mice were exposed to a 12L: 12D photoperiod (L: 06:00 h– 18:00 h) and the room was illuminated at daytime by white fluorescent lights (illuminance was approximately 400 lux).

All animals were euthanized using an overdose of halothane (Zeneca, Johannesburg, South Africa) and then perfused transcardially with 0.9% saline followed by 4% paraformaldehyde (PFA; Saarchem, Johannesburg, South Africa) in phosphate-buffered saline (PBS, 0.1M). The heads were removed and post fixed in 4% PFA for approximately 4 hours before being transferred to 0.5% PFA diluted in PBS. Some animals were perfused by Zamboni’s fixative (phosphate buffered picric acid-formaldehyde, PAF) and their heads and eyes were stored in the same fixative. Each eye was marked dorsally for orientation, enucleated, cut at the orra serrata, the lens and vitreous body were removed. The retina was carefully dissected from the eye cup and stored in PBS with 0.05% sodium azide at 4°C until further processing.

Immunocytochemistry was performed on unsectioned whole retinae or retinal sections. Vertical sections from several retinae were immunolabelled to validate the presence of the photoreceptor types and to test the specificity of the antibodies in the two species. Retinal quadrants were cryoprotected in 30% sucrose, embedded in Jung Tissue Freezing Medium (Leica Instruments, Germany) and sectioned vertically (i. e. perpendicular to the retinal layers) at a thickness of 14 μm with a cryostat and mounted on Superfrost Plus slides (ThermoFisher Scientific). Retinal whole mounts were used to study the topographical distribution and densities of the photoreceptors. For each species, one whole retina was used per analysis of each photoreceptor type. Whole retinae were sequentially incubated in phosphate buffer containing 10%, 20% and 30% sucrose for cryoprotection and then repeatedly shock-frozen and thawed to improve penetration of the antibodies. Whenever patches of the pigment epithelium remained attached to the retina, the retinae were incubated before cryoprotection in a solution comprising of 5 ml 1.8% saline (NaCl in H_2_O), 4 ml 30% H_2_O_2_, 1 ml H_2_O and one drop of a 25% NH_3_ solution (modified from [29,30]), until the remaining pigment epithelium were bleached. The bleaching does not affect the subsequent immunoreactions [31].

### Antibodies

Primary antibodies used in this study and their dilutions are listed in Table 1. In *M*. *namaquensis*, three mouse monoclonal antibodies namely Rho4D2, OS-2 and COS-1 labelled rods, S-cones and M/L-cones, respectively. These antibodies were used for assessment of photoreceptor distributions and densities. A rabbit polyclonal antibody JH455 and a mouse monoclonal antibody COS-1 were used for double labelling of S- and M/L-cones, respectively. In *R*. *pumilio*, the OS-2 labelled S-cones, a rabbit polyclonal antibody (AB5405) labelled M/L-cones, and a rat polyclonal antibody (AO) labelled rods. The OS-2 and the AB5405 were used also for S- and M/L-cones double labelling. For the labelling of the ipRGCs in both species, a rabbit polyclonal antibody (PA1-780) was used.

**Table 1:** List of primary antibodies used in the present study.

Species/Specifity	*Micaelamys namaquensis*	*Rhabdomys pumilio*	Immunogen
Rods	Mab; Rho4D2 (1:400*)^a^	Pab; AO (1:100)^b^	Rhodopsin N terminal [32,33]
M- and L-cones	Mab; COS-1 (1:50)^b^	Pab; AB5405 (1:250*)^c^	M/L-pigment C terminal [33]
S-cones	Mab; OS-2 (1:5000)^b^	Mab; OS-2 (1:5000)^b^	S-pigment C terminal [33]
	Pab; JH455 (1:5000)^d^		
ipRGCs	Pab; PA1-780 (1:200*)^e^	Pab; PA1-780 (1:200*)^e^	Melanopsin C terminal [4]

Abbreviations: Mab, monoclonal antibody; Pab, polyclonal antibody.

*Half concentrations were used for vertical sections.

^a^Abcam

^b^kindy provided by Ágoston Szél

^c^Millipore

^d^kindy provided by Jeremy Nathans

^e^Thermo Scientific.

### Immunocytochemistry

Whole retinae were processed free floating, and retinal sections mounted on slides. In order to block nonspecific binding sites, tissue was pre-incubated in either 1% bovine serum albumin or a blocking solution comprising 10% normal goat serum or normal donkey serum (NGS/NDS), depending on the antibody to be used, and 3% Triton in PBS for 30 minutes. For single immunolabelling of rods, S-cones, M/L-cones and ipRGCs, the tissue was then incubated in a solution comprising of the primary antibody diluted in PBS with 3% NDS/NGS, 0.3% Triton X-100 and 0.05% sodium azide for 3–7 days (whole retinae) or overnight (sections) at room temperature. The following day the tissue was washed in 0.3% Tween 20 diluted in PBS (PBST). In most whole mounts, the binding sites of the primary antibodies were visualized by a peroxidase anti-peroxidase reaction in order to obtain a permanent label for quantitative analysis using light microscopy. In this case incubation with the primary antibody was followed by a 90 min incubation with a biotinylated goat anti-rabbit IgG, goat anti-mouse IgG or donkey anti-rat IgG, respectively. Subsequent incubation was in avidin-peroxidase complex (Vectastain® Elite® ABC-Kit, Vector Laboratories Inc., Burlingham, USA). The peroxidase reaction was developed in a PB solution containing diaminobenzidine tetrahydrochloride (0.05%), nickel ammonium sulphate (0.01%), cobalt chloride (0.0125%) and hydrogen peroxide (0.02%). After the DAB reaction, whole retinae were flattened onto slides with the photoreceptor side up, semi-dried for better adhesion and coverslipped with an aqueous mounting medium (AquaPoly/Mount, Polysciences Inc., Warrington, PA, USA).

For some whole mounts and all sections, the binding sites of the primary antibodies were visualized by immunofluorescence. In most cases, the tissue was incubated for 1 h in the appropriate secondary antibody conjugated with either Alexa 594 (red fluorescence) or Alexa 488 (green fluorescence; dilution 1:500 to 1:1000; ThermoFisher Scientific). For the visualization of the bound monoclonal antibodies OS-2 and COS-1, the retinae were incubated in a biotinylated anti-mouse IgG antibody for 12–24 hours and subsequently with either Texas Red avidin D conjugate (50 μg/ml, Vector Laboratories) or streptavidin Alexa Fluor® 594 conjugate (100 μg/ml, Molecular Probes, Eugene). Double-labelling was performed by incubating the tissue in a mixture of the respective antibodies in the same medium as stated above. The binding sites of the primary antibodies were then visualized by an appropriate mixture of the above mentioned secondary antibodies. Peanut agglutinin (5 μg/ml, Sigma; PNA), lectin isolated from *Arachis hypogea*, was used as a marker of all cones. The binding of biotinylated PNA was detected with either avidin D conjugated either to Texas Red or to AMCA (100 μg/ml, Vector Laboratories). After the immunocytochemical staining, whole retinae were flattened onto slides with the photoreceptor side up, semi-dried and coverslipped with the AquaPoly/Mount. The same mounting medium was used to coverslip the sections. The labelled slides were stored in a fridge until inspection.

### Data capture and photoreceptor density analyses

The immunostained retinae were analyzed with an Olympus BX51 or Zeiss Axiophot microscope equipped with differential interference contrast and epifluorescence with appropriate filter settings. Micrographs were taken with a CCD camera (Olympus DP50, or SPOT RT Slider 2.3.1.1, Diagnostic Instruments, Inc.) and SPOT Advanced 5.0 software (Visitron Systems, Puchheim, Germany). The images were adjusted for brightness and contrast using Image J software (National Institute of Health, USA).

For rod and cone cell counting, a grid was superimposed over the whole mounted retina, dividing it into 1 mm^2^ squares. At 1000x magnification, one photo (117 x 88 μm) was captured for each square and the number of the respective cells was counted within the micrograph. Because the cell densities of the photoreceptors were high and the outer segments long, counting was carried out manually. The counts were then standardized to indicate the number of photoreceptors per mm^2^. Cone densities were not corrected for shrinkage, because tissue shrinkage was negligible in whole retinae mounted with the aqueous medium. Vector illustrations for whole retinae were created using Adobe Illustrator CS5 (Adobe Systems Inc., CA) and the density values plotted in to create topographical maps. Camera lucida drawings were made for ipRGCs and the cells were counted directly. Several ratios between the photoreceptor classes were determined using the mean densities of the photoreceptors (per mm^2^) that were calculated for either the central and peripheral retinal regions or the whole retina. The recognition of ipRGCs as a photoreceptor class is relatively recent, and thus the majority of published studies typically refer to the collection of rods and cones as the total photoreceptor population. In order to avoid confusion, a distinction is made in the present study between the total photoreceptor population (TPP) and the total visual-photoreceptor population (TVPP), of which the latter excludes the ipRGCs. Standard errors were indicated where applicable.

## Results

Both studied species have eyes that are laterally oriented. Their eye dimensions are summarized in Table 2. Although both species have a similar body size, the nocturnal *M*. *namaquensis* has significantly larger eye than the diurnal/crepuscular *R*. *pumilio*. The former species also has a larger lens relative to the eye size. In *M*. *namaquensis*, the lens has a diameter (calculated as the average between the measured axial length and the equatorial diameter) of 0.68 times the eye diameter. In *R*. *pumilio*, this ratio is 0.55.

**Table 2:** Size of the eyeball and the lens^1^.

	Eye				Lens	
Species	Axial length	Equatorial diameter	Corneal diameter^2^	Height of the frontal segment^3^	Equatorial diameter	Axial length
*Rhabdomys pumilio*	4.13±0.07	4.43±0.14	3.51±0.06	1.64±0.04	2.54±0.09	2.14±0.27
*Micaelamys namaquensis*	5.98±0.02	5.83±0.17	5.05±0.34	2.41±0.11	4.27±0.20	3.77±0.18

^1^Dimensions given in millimeters and expressed as mean value ± standard deviation

^2^measured as diameter of the eyeball in the sclero-corneal passage

^3^measured as distance between polus anterior bulbi and sclero-corneal passage.

**Fig 1:**
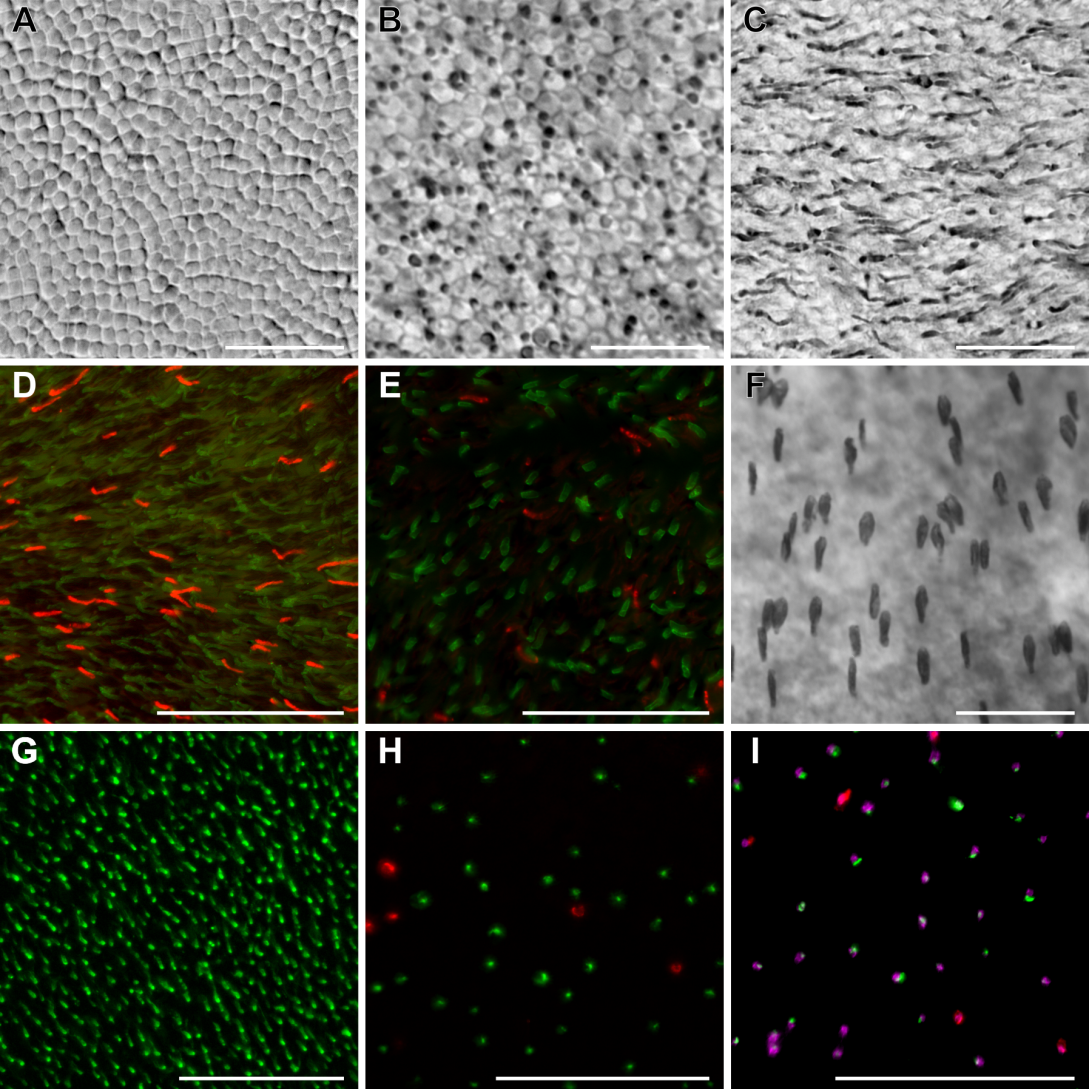
Identification of rods and cones in flat mounted retinae of *Rhabdomys pumilio* (A–F) and *Micaelamys namaquensis* (G–I). (A) The retinal photoreceptor mosaic at the level of the inner segments. (B, C) Rod opsin labelled with AO antibody (mid-peripheral retina), the focus is on the transition between rod inner and outer segments (B), and rod outer segments (C). (D, E) Double immunofluorescence labelling of M/L cone opsin (in green) and S-cone opsin (in red). Note that cone densities are much higher in the ventral part of central retina (D) when compared to peripheral retina (E). (F) Higher power photomicrograph showing S-cone densities in central retina. Identification of photoreceptors in *M*. *namaquensis* (G–I). (G) Rod opsin labelled with RhoD2 antibody (peripheral retina), the focus is on rod outer segments. (H) Double immunofluorescence labelling of M/L cone opsin (in green) and S-cone opsin (in red) in central retina. (I) Triple immunofluorescence labelling of all cones (PNA labelling, in magenta), M/L-cone opsin (in green) and S-cone opsin (in red) in the central retina. Note that dual cones were not detected in either of the two species and that *R*. *pumilio* has much higher cone densities than *M*. *namaquensis*. Scale bars; 10 *μ*m (A–C, F), 50 *μ*m (D, E, G–I).

**Fig 2:**
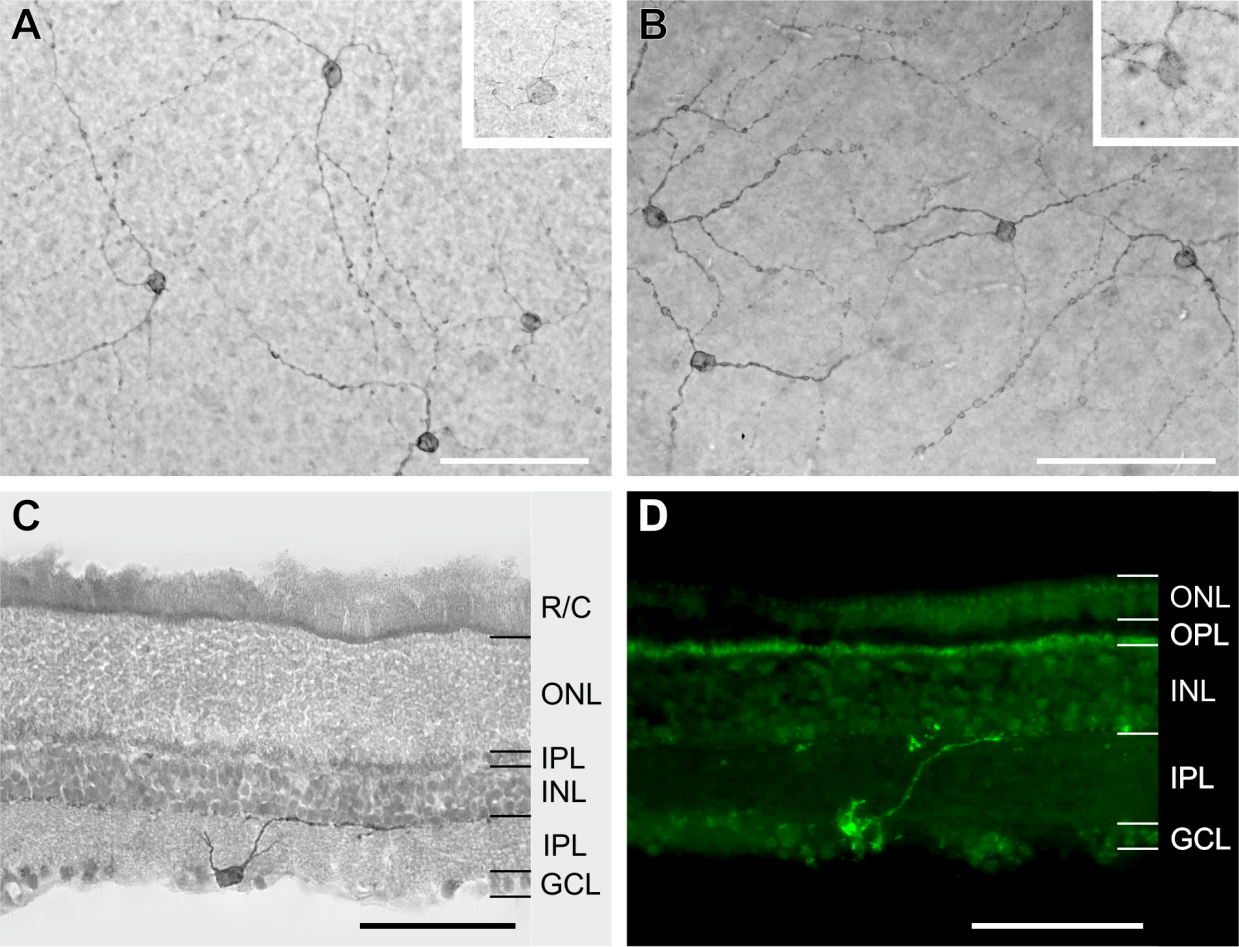
Immunolabelling of melanopsin positive ganglion cells in *Micaelamys namaquensis* (A, C) and *Rhabdomys pumilio* (B, D). (A, B) Flattened retinae showing ipRGCs dendritic arbors with beaded varicosities. The upper right insets show putative M2 type ipRGCs, which were faintly labelled and had larger somata. (C, D) Vertical sections of the retina demonstrating that the somata of ipRGCs are situated in the GCL with dendrites stratifying in the outermost margin (OFF sublamina) of the IPL. GCL, ganglion cell layer; INL, inner nuclear layer; IPL, inner plexiform layer; ONL, outer nuclear layer; OPL, outer plexiform layer; R/C, outer segments of rods and cones. The binding sites of the primary antibodies were visualized by a peroxidase anti-peroxidase reaction (diaminobenzidine staining) in (A–C) and by Alexa 488 conjugated secondary antibody in (D). Scale bars; 100 μm.

Immunolabelling validated the presence of four photoreceptor populations in *R*. *pumilio* and *M*. *namaquensis*: rods, S-cones, M/L-cones as well as ipRGCs (Figs 1 and 2). Dual cones, i.e. cones coexpressing S- and M/L-opsins, were not detected in either of the two species (Fig 1D, 1E, 1H and 1I). With the exception of the relatively uniform distribution of rods and ipRGCs in the retina of *M*. *namaquensis*, all of the photoreceptor classes in both species displayed some topographical heterogeneity. The retinas of both species were characterized by high rod densities but interestingly, cone density in the retina of *R*. *pumilio* was nearly as high as the density of rods. From the mean photoreceptor densities (per mm^2^) that were calculated for the retina as a whole (not considering topographical layout), cones made up to ~44.9% of the TVPP in *R*. *pumilio* and only ~7.5% in *M*. *namaquensis*. Despite this high density of both rods and cones in *R*. *pumilio*, the mean ratio of cones to rods (1:1.23) fell within the typical range, yet at the higher end, which has been reported for many diurnal small mammals [3]. In *M*. *namaquensis*, the mean cone to rod ratio was 1:12 and was also as expected for a nocturnal mammal. When the cone/rod ratios were compared between central and peripheral retinae, it was slightly higher centrally (1:1.49) than peripherally (1:1.32) in *R*. *pumilio*. Note that these values are slightly above the overall mean cone/rod ratio due to the non-overlapping topographical density layouts (see below). Cone/rod ratios were lower centrally (1:10.5) than peripherally (1:14.1) in *M*. *namaquensis*. In stark contrast to the conventional photoreceptors, ipRGCs were very sparsely distributed and constituted approximately 0.024% of the TPP for both species. The mean ipRGC/rod and ipRGC/cone ratios were 1:2280 and 1:1861 in the diurnal species and, 1:1390 and 1:316 in the nocturnal species.

### Rod photoreceptors

**Fig 3:**
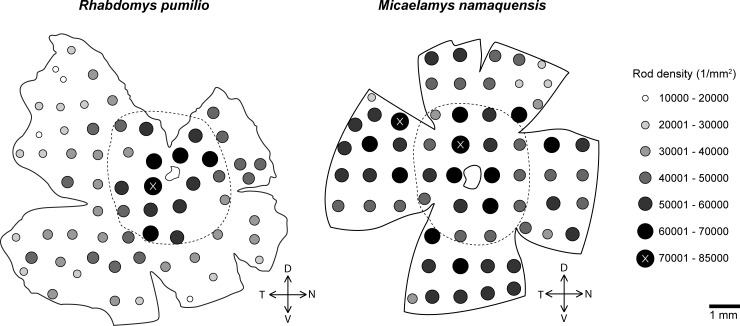
Topographical map of the rod density distribution in *Rhabdomys pumilio* (left) and *Micaelamys namaquensis* (right). The filled circles indicate rod density; the irregular outline that is more or less in the center of the retina indicates the position of the optic nerve head. The dashed line separating the central retina from the peripheral retinal regions designates the areas that were used to calculate photoreceptor ratios between the central and the peripheral retina. D, dorsal; V, ventral; T, temporal; N, nasal.

Rods were labelled with rhodopsin specific antibodies AO (*R*. *pumilio*) and Rho4D2 (*M*. *namaquensis*) and had the highest densities (per mm^2^) of all photoreceptor classes (Fig 1C and 1G). In the *R*. *pumilio* retina, rod density was highest in the central region (mean: 56618 rods/mm^2^) and steadily decreased towards the periphery (mean: 32689 rods/mm^2^); see Fig 3. The density at 84467 rods/mm^2^ in the ventro-temporal region close to the optic nerve and was lowest at the far ventro-nasal periphery (14739 rods/mm^2^). Despite some heterogeneity in topographical distribution of rod densities, no distinct cento-peripheral gradient was observed in the retina of *M*. *namaquensis*. Density values ranged between 20712 rods/mm^2^ and 81088 rods/mm^2^ (Fig 3). Although the values at the densest regions were nearly the same in the two species, the overall mean density was higher in the nocturnal (50820 rods/mm^2^) compared to the diurnal species (38766 rods/mm^2^).

### Cone photoreceptors

**Fig 4:**
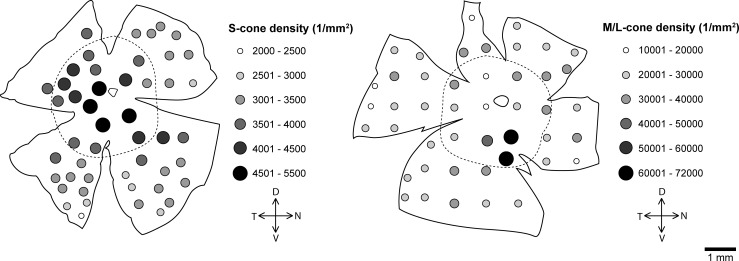
Topographical map of the S-cone (left) and M/L-cone density distribution (right) in *Rhabdomys pumilio*. The filled circles indicate cone density; the irregular outline that is more or less in the centre of the retina indicates the position of the optic nerve head. The dashed line separating the central retina from the peripheral retinal regions designates the areas that were used to calculate photoreceptor ratios between the central and the peripheral retina. D, dorsal; V, ventral; T, temporal; N, nasal.

**Fig 5:**
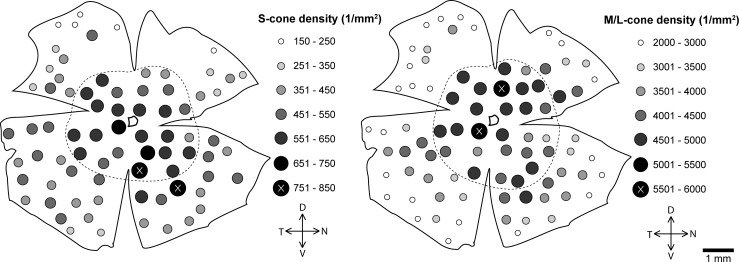
Topographical map of the S-cone (left) and M/L-cone density distribution (right) in *Micaelamys namaquensis*. The filled circles indicate cone density; the irregular outline that is more or less in the centre of the retina indicates the position of the optic nerve head. The dashed line separating the central retina from the peripheral retinal regions designates the areas that were used to calculate photoreceptor ratios between the central and the peripheral retina. D, dorsal; V, ventral; T, temporal; N, nasal.

The overall mean cone density was almost 8 times higher in *R*. *pumilio* (31640 cones/mm^2^) than in *M*. *namaquensis* (4113 cones/mm^2^). Two separate cone populations were identified (Fig 1D, 1E, 1H and 1I). OS-2 positively labelled short wave sensitive opsins in both species while red/green sensitive opsins were labelled using the AB5405 antibody in *R*. *pumilio* and COS-1 in *M*. *namaquensis*. Both species had M/L-cone dominant retinas where M/L-cones made up ~89% of the total cone population. In *R*. *pumilio*, M/L-cones had an overall mean density of 28302 M/L-cones/mm^2^. Topographically M/L-cones did not form a centro-peripheral gradient, but were densely concentrated in a spherical area located about 0.75–1.5 mm ventral to the optic disc where the density peak was 71319 M/L-cones/mm^2^ (Fig 4). Beyond this very dense area, the M/L-cones were distributed relatively uniformly (25920 M/L-cones/mm^2^), yet the region with the lowest count (16218 M/L-cones/mm^2^) was located at the furthest periphery. Unlike the M/L-cones, the S-cones displayed a centro-peripheral gradient, but with a slight density streak directed in a dorso-temporal to ventro-nasal direction (Fig 4). S-cone density ranged from 2401 to 5019 S-cones/mm^2^. In *M*. *namaquensis*, both cone types displayed a centro-peripheral density gradient. The minimum and maximum values ranged from 2018 to 5066 M/L-cones/mm^2^, whilst that for the S-cones from 151 to 800, respectively (Fig 5). Naturally, the ratio between the two cone populations varied between different regions of the retina. Using the density means of central and peripheral regions, the S-cone to M/L-cone ratios were estimated to be approximately 1:7.8 in central and peripheral regions of *M*. *namaquensis* as well as in the central region of *R*. *pumilio*. This ratio was slightly lower (1:6.8) in the peripheral region of the latter species and much higher (1:15) when it is calculated only in the spherical region where the M/L-cones are highly concentrated (Figs 4 and 5).

### Intrinsically photosensitive ganglion cells (ipRGCs)

**Fig 6:**
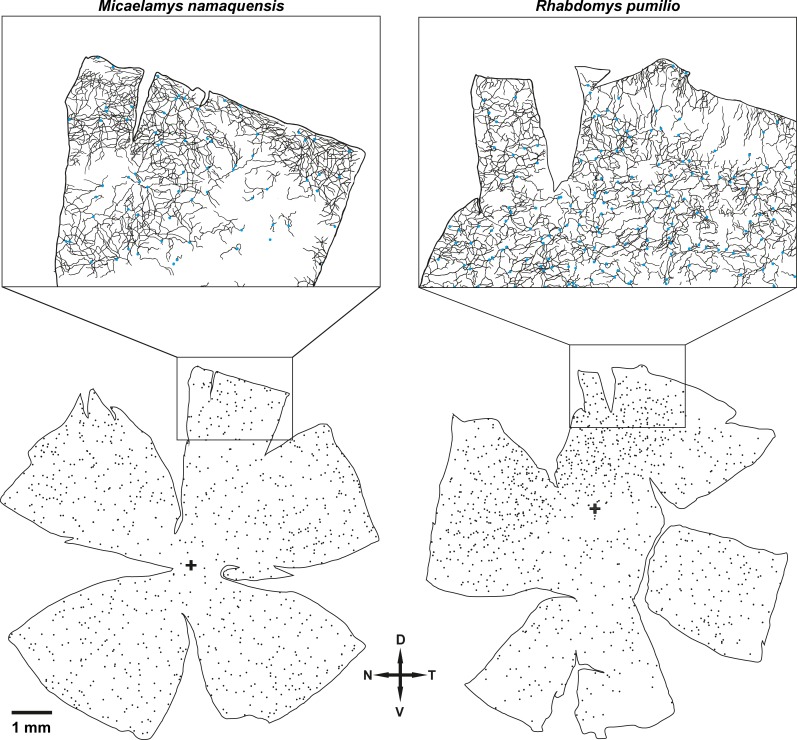
Topographical distribution of melanopsin positive retinal ganglion cells (ipRGCs) in whole mounted retina of *Micaelamys namaquensis* (left) and *Rhabdomys pumilio* (right); each dot represents one ipRGC. The enlarged retinal segments (in the top two rectangles) display camera lucida drawings of the photoreceptive nets that are formed the ipRGCs and their overlapping dendrites; the beaded appearance of the dendrites is not shown.

In both species, we used a rabbit polyclonal antibody, PA1-780, that recognizes the C-terminus of melanopsin to label the ipRGCs. Compared to rods and cones, the amount of positively labelled ipRGCs was very low, while *R*. *pumilio* had a mean of 17 ± 14 ipRGCs/mm^2^ and a total of 1012 ipRGCs across the entire retina, *M*. *namaquensis* had a mean of 13 ± 6 ipRGCs/mm^2^ and a total of 862 ipRGCs across the entire retina. The density distributions of the ipRGCs differed between the two species (Fig 6). In *R*. *pumilio*, the ipRGCs were concentrated in the dorso-nasal quadrant where the density peak was 56 ipRGCs/mm^2^. Across the remaining retinal surface the ipRGCs were uniformly distributed (~11–20 ipRGCs/mm^2^). In the retina of *M*. *namaquensis*, the ipRGCs appeared to be uniformly distributed across the retina. However, slightly lower densities (~10–15 ipRGCs/mm^2^) were observed in the central retina and slightly higher densities in the dorsal retina (Fig 6). Immunolabelled ipRGCs in whole mounted retinae revealed the photoreceptive net and a beaded appearance of the dendrites (Fig 2A and 2B). In both species, the vertical sections used for testing whether PA1-780 recognized melanopsin in the two study species displayed M1 type ipRGCs with somata located in the ganglion cell layer (GCL) and their dendrites stratifying the outermost margin (OFF sublamina) of the inner plexiform layer (IPL; Fig 2C and 2D). In addition to darkly labelled ipRGCs with discrete dendritic arbors, a subpopulation of weakly stained ipRGCs with somewhat larger somata and poorly labelled dendritic arbors were observed in whole mounted retinae (Fig 2A and 2B). Some of these putative M2 type ipRGCs were barely detectable.

### Retinal magnification factor (RMF) and inter-receptor angle

We have estimated retinal magnification factors (RMF) using the following formula published by [34]; RMF = 2π × PND / 360, where PND is posterior nodal distance, which can be calculated from axial eye ball length (AEL). For a nocturnal eye, PND = 0.52 × AEL, for a crepuscular eye, PND = 0.57 × AEL. The RMF for *R*. *pumilio* is 0.041 and for *M*. *namaquensis* 0.054, i.e., the distance on the retina that subtends one degree is 41 *μ*m and 54 *μ*m, respectively. Assuming a square array of photoreceptors, the number of photoreceptors per linear degree of visual angle was calculated as the square root of the maximal photoreceptor density. The peak cone density (both cone types counted) was ~ 76338/mm^2^ for *R*. *pumilio* and ~ 5766/mm^2^ for *M*. *namaquensis*, the peak rod densities are indicated above. The retina of *M*. *namaquensis* harbors four cones and 15 rods per degree, i.e., a minimum inter-cone angle is 0.243°, a minimum inter-rod angle 0.065°. The retina of the diurnal/crepuscular *R*. *pumilio* contain 11 cones and 12 rods per degree, i.e., a minimum inter-cone and inter-rod angles are 0.088°and 0.084°, respectively.

## Discussion

Most sensory systems display adaptations to the specific lifestyle of a species. Adaptations of the visual system range from the anatomical (e.g. eye size, shape, and positioning on the animal’s head) to the physiological adaptations (e.g. the arrangement of retinal circuitry and photoreceptor topographies) [16,26,35–37]. In the current study, we present and compare the size of the eyes, the density and topographical distributions of rods, cones and ipRGCs in two murid rodents that inhabit different photoenvironments; the diurnally active *Rhabdomys pumilio* and the nocturnally active *Micaelamys namaquensis*.

The sizes of the eyes of both studied species are well within the range of eye dimensions in surface-dwelling rodents of similar body size [38]. Nevertheless, the eye of nocturnal *M*. *namaquensis* was approximately 40% larger than that of diurnal/crepuscular *R*. *pumilio*. Moreover, the lens was larger in the nocturnal species, in both absolute and relative terms. A corollary of large eye is a large pupil, which enhances the photon flux to the retina. A large, almost spherical lens provides a short focal length, which, combined with a wide pupil, gives the eye high light gathering power. These features are characteristic of nocturnal mammals adapted to low light levels [39]. The relative size of the lens observed in *R*. *pumilio* is typical for arrhythmic or crepuscular mammals rather than for truly diurnal mammals adapted to bright daylight [39].

We report rod-dominated duplex retinas in *R*. *pumilio* and *M*. *namaquensis*. This is a typical feature of mammalian retinae, irrespective of whether a species is nocturnal or diurnal [3,16]. As expected, cone to rod ratios were in line with the daily activity rhythms of the study species. The high cone density value (~44% of the TVPP) and the low ratio of cones to rods (1:1.23) observed in *R*. *pumilio* are comparable, but slightly lower in other diurnal muroid rodents [13,18,19,40]. Despite being fundamentally diurnal, *R*. *pumilio* is active mostly around dawn and dusk [28]. Therefore, a high density of both cones and rods most likely provides this species with adequate vision, even at dawn and dusk when the photoenvironment changes substantially. This proportion of rods and cones may also improve vision during the daytime when mice are making use of darker areas, such as underground burrows or nests. In *M*. *namaquensis*, only ~7.5% of the TVPP is comprised of cones and the overall mean cone to rod ratio (1:12.4) was high compared to that of *R*. *pumilio*. These values are unusually high when compared to other nocturnal murids where cones comprise 0.3–3% of the TVPP [20,21,23,41,42].

Despite having similarly high density peak values, *M*. *namaquensis* had on average ~12000 more rods per mm^2^ than *R*. *pumilio* due to different topographical arrangements. Research suggests that retinal specializations (e.g. areae centrales, foveae and visual streaks; [24]) enhance vision in specific parts of the visual field, but studies on murid retinae have more often than not focused solely on cone and ganglion cell topographies. Notably, some species may also have unique rod arrangements. In the California ground squirrel *Spermophilus beecheyi* for instance, rods are distinctively distributed across the dorso-ventral axis and have a density peak in the ventral retina [17]. Although the functional significance of such a rod mosaic lacks a conclusive explanation, it may point to the species’ visual needs in the burrow environment [17]. The present study reports that rods follow a nearly homogeneous spatial distribution in the retina of *M*. *namaquensis* and a sharp centro-peripheral density gradient (area centralis) in the retina of *R*. *pumilio*. As *M*. *namaquensis* is almost exclusively active by night [43], its vision is largely mediated by rods and a homogeneous distribution should contribute to increased visual acuity across the visual field. Photoreceptor distributions often form areae centrales in animals that inhabit closed or complex environments [24]. For this reason, the centro-peripheral rod density gradient in *R*. *pumilio* might be functionally significant when moving in structured or closed areas with low lighting conditions during the day (e.g. underground burrows or nests).

In both study species, immunocytochemistry revealed two cone populations that each expressed either S-opsin or M/L-opsin. Therefore, as in most mammals, dichromacy appears to be the prevalent mode of color vision in *R*. *pumilio* and *M*. *namaquensis*. Monochromacy has only been identified in a few rodents, such as the pygmy field mouse (*Apodemus microps*), African giant rats (*Cricetomys gambianus* and *C*. *emini*) and the Syrian golden hamster (*Mesocricetus auratus*) [23,44,45]. Mammals display a large variety of topographical density arrangements of cones [3]. Many smaller terrestrial mammals possess a ventral bias of S-cones and in some cases a complete separation between these two spectral cone types [25,26,31]. Although such a pattern was not observed in either of the study species, cone arrangements were nonetheless heterogeneous. In *M*. *namaquensis*, both cone densities decreased gradually from the center towards the periphery and roughly consistent S-cone to M/L-cone ratios were observed across the retina. Many other species with heterogeneous cone distributions typically show centro-peripheral density gradients (e.g. see [44,46]). This was also true for S-cones in *R*. *pumilio*, except that a slightly flatter distribution (resembling a visual streak) was observed. In contrast, the densely populated area of M/L-cones inferior to the optic nerve was without a centro-peripheral gradient. This specialized area might imply that spectral sensitivity is greater in the central, somewhat upper, visual field. Interestingly, in the strictly diurnal Californian ground squirrel, cones constitute 86% of the TVPP and have a peak value of 49550/mm^2^ [17]. This value is one and a half times lower than just the M/L-cone density peak value (~71319/mm^2^) in the specialized area of *R*. *pumilio*. Although the ratio of S-cones to M/L-cones in *R*. *pumilio* depended on the retinal region, the overall mean values for the two species (*R*. *pumilio*: 1:8.5; *M*. *namaquensis*: 1:7.8) were in line with those reported in most small mammals. Generally, it is around 1:10 irrespective of whether the species has a rod dominant or cone dominant retina [25,26]. Most unusually, the opposite pattern has been observed in some mole-rats belonging to the family Bathyergidae, where the majority of immunostained cones expressed S-opsins [47]. This pattern, however, seem not to have an adaptive value and appears to be a mechanistic consequence of naturally low levels of thyroid hormones [48,49].

Eye size determines the image size on the retina and photoreceptor densities limit resolution with which the image is sampled. From our calculations for eye size, RMF and inter-receptor angle, it can be concluded that in strictly nocturnal *M*. *namaquensis* rods provide much finer image sampling than cones, whereas in *R*. *pumilio*, which is mostly active during the day but also at dusk and dawn, both photoreceptor types provide fine image sampling. However, the peak photoreceptor densities cannot be interpreted in terms of visual acuity. The density of ganglion cells, not the density of the photoreceptors, limits visual acuity in rodents, because commonly there is a considerable convergence from photoreceptors to ganglion cells (reviewed in [50]).

Melanopsin expressing ipRGCs constitute a small subpopulation of the total retinal ganglion cell population (1–2%) [4]. In the present study, immunolabelling likewise exposed very sparse populations of ipRGCs in *R*. *pumilio* (~17/mm^2^) and in *M*. *namaquensis* (~13/mm^2^), but their spatial distributions were distinctly different. In *R*. *pumilio*, ipRGCs were most dense in the dorso-nasal retina and with a density gradient not corresponding to either rod or cone density topographies. In *M*. *namaquensis*, ipRGC distribution was as homogeneous as that of its rods. Presently, studies have identified five distinct ipRGC subtypes (M1 through M5); they differ in soma size, dendritic morphology, the location of their dendritic stratification, the strength of their intrinsic responses, the brain regions to which they project etc. [51,52]. Recent studies have demonstrated that subtypes M3–M5 cannot be reliably identified by immunolabelling due to low expression levels of melanopsin [53,54]. Therefore, the ipRGCs identified in this study were most likely M1 and M2 types. From the vertical sections it could be seen that somata of the PA1-780 labelled cells resided in the GCL with their dendrites stratifying the outermost margin (OFF sublamina) of the IPL. This is characteristic of the M1 type [4,55]. Although we did not inspect enough vertical sections to be able to confirm it, the faintly labelled ipRGCs with larger somata were likely M2 cells. Because some of these putative M2 cells were barely discernible, we cannot exclude that the total counts of the ipRGCs is underestimated. The M2 cells with melanopsin expression levels beneath immunocytochemical detection threshold have been reported in the rat [54]. Interestingly, some evidence suggests that the ratio between M1 and M2 cell types could potentially contribute to differences in the temporal niches of species and that M1 cells are more predominant in diurnal than nocturnal species [56]. Whether this applies to *R*. *pumilio* and *M*. *namaquensis* remains to be tested.

## Conclusions

The presence and spatial distribution of rods, cones and ipRGCs in *R*. *pumilio* and *M*. *namaquensis* retinae are described in the present study. The results indicate that both species are dichromats, with rods and ipRGCs being the most and the least abundant photoreceptor classes, respectively. Cone to rod ratios seem to reflect the temporal niches of the two species and regional specializations of the photoreceptors were more evident in *R*. *pumilio* retina, compared to *M*. *namaquensis* retina. Although the topographical distributions of the photoreceptors are likely related to the specific visual needs of the species in their environments, the exact functional significance thereof, especially for ipRGCs, needs further investigation.
